# A Physical Fatigue Evaluation Method for Automotive Manual Assembly: An Experiment of Cerebral Oxygenation with ARE Platform

**DOI:** 10.3390/s23239410

**Published:** 2023-11-26

**Authors:** Wanting Mao, Xiaonan Yang, Chaoran Wang, Yaoguang Hu, Tianxin Gao

**Affiliations:** 1 Department of Civil and Environmental Engineering, Imperial College London, London SW7 2AZ, UK; w.mao23@imperial.ac.uk; 2 School of Mechanical Engineering, Beijing Institute of Technology, Beijing 100081, China; chaoranwang@bit.edu.cn (C.W.); hyg@bit.edu.cn (Y.H.); 3 Key Laboratory of Industry Knowledge & Data Fusion Technology and Application, Ministry of Industry and Information Technology, Beijing Institute of Technology, Beijing 100081, China; 4 Yangtze Delta Region Academy, Beijing Institute of Technology, Jiaxing 314019, China; 5 School of Life Science, Beijing Institute of Technology, Beijing 100081, China; gtx@bit.edu.cn

**Keywords:** physical fatigue, automotive manual assembly, cerebral oxygenation, metabolic energy consumption

## Abstract

Due to the complexity of the automobile manufacturing process, some flexible and delicate assembly work relies on manual operations. However, high-frequency and high-load repetitive operations make assembly workers prone to physical fatigue. This study proposes a method for evaluating human physical fatigue for the manual assembly of automobiles with methods: NIOSH (National Institute for Occupational Safety and Health), OWAS (Ovako Working Posture Analysis System) and RULA (Rapid Upper Limb Assessment). The cerebral oxygenation signal is selected as an objective physiological index reflecting the human fatigue level to verify the proposed physical fatigue evaluation method. Taking auto seat assembly and automobile manual assembly as an example, 18 group experiments were carried out with the ARE platform (Augmented Reality-based Ergonomic Platform). Furthermore, predictions of metabolic energy expenditure were performed for experiments in Tecnomatix Jack. Finally, it is concluded that the proposed physical fatigue evaluation method can reflect the human physical fatigue level and is more accurate than the evaluation of metabolic energy consumption in Tecnomatix Jack because of the immersion that comes with the AR devices and the precision that comes with motion capture devices.

## 1. Introduction

Assembly and manufacturing processes usually involve a mass of flexible and elaborate manual operations performed by human operators working in the workshop. Especially in the automotive assembly industry, there is much assembly work that is done manually by workers, and these assembly tasks, including lifting, handling, and installation, are usually characterized by high frequency and high load [1]. Long hours of high workload can easily lead to worker fatigue, physical strength decline, and even cause injury and disease [2]. Workers in a fatigued state easily have low efficiency and more operational errors, which would have a negative impact on both company revenue and workers’ physical health [3]. These problems can only be avoided if ergonomics are properly applied so that workers are able to perform assembly work within their physical capabilities. Therefore, accuracy in evaluating workers’ fatigue is very crucial [4].

Energy expenditure is an important factor in physical fatigue because muscle contraction during exercise uses up stored energy in the body [5,6]. When people are in a state of fatigue, the internal catabolism and anabolism of the body are difficult to maintain in balance, causing muscle contractions to become weak, inhibiting the central nervous system, and finally exhausting the whole body [7]. By decomposing assembly movements, acquiring movement parameters, and calculating human fatigue assessment based on energy consumption, the fatigue level of workers performing assembly work can be assessed [8]. Tecnomatix Jack 8.0 is a human modeling and simulation software developed by Siemens Digital Industries Software (The company is headquartered in Plano, TX, USA), that is a powerful tool for human-centric design and ergonomic analysis. It aids in creating work environments that are not only efficient and productive, but also considerate of human factors to enhance safety and well-being. However, the traditional energy-based physical fatigue assessment, such as Tecnomatix Jack, has disadvantages, such as the influence of individual differences and the influence of external environmental factors, so it cannot assess human fatigue more accurately [9]. Especially for the prediction of energy expenditure in Tecnomatix Jack, its prediction accuracy cannot reach a high level due to limitations in model accuracy, assumptions about static environments, insufficient individual differences, and inaccurate modeling of complex actions, [10,11].

The brain is a highly energy-consuming organ, consuming 20% of the body’s energy at about 2% of the body’s weight, and can be irreversibly damaged by brief periods of hypoxic conditions [12]. The rapid consumption of energy by the brain during exercise can lead to changes in cerebral oxygen saturation, thus cerebral oxygen saturation can be used to evaluate physical fatigue [13,14]. Cerebral oxygen saturation is usually measured by near-infrared spectroscopy (NIRS). NIRS cerebral oxygen saturation monitoring quantifies the relative concentrations of oxyhemoglobin and deoxyhemoglobin within a target tissue by relying on the transmission and absorption of near-infrared light through the tissue, allowing for an estimate of the balance between cerebral oxygen supply and demand [13]. Matsuura et al. studied changes in brain and muscle oxygenation in humans during static and dynamic knee extension to voluntary fatigue, and used cerebral oxygen during exercise to assess the level of cerebral reoxygenation and thus the degree of fatigue [15]. Monroe et al. explored the changes in fatigue level and cerebral oxygenation produced by exercise at different intensities, and used cerebral oxygenation to explain the changes in fatigue and energy during exercise recovery [16]. Hiura et al. analyzed cerebral oxygenation in rowing athletes, which varied at different exercise intensities and fatigue levels, thus assessing the level of fatigue [17].

**Figure 1:**
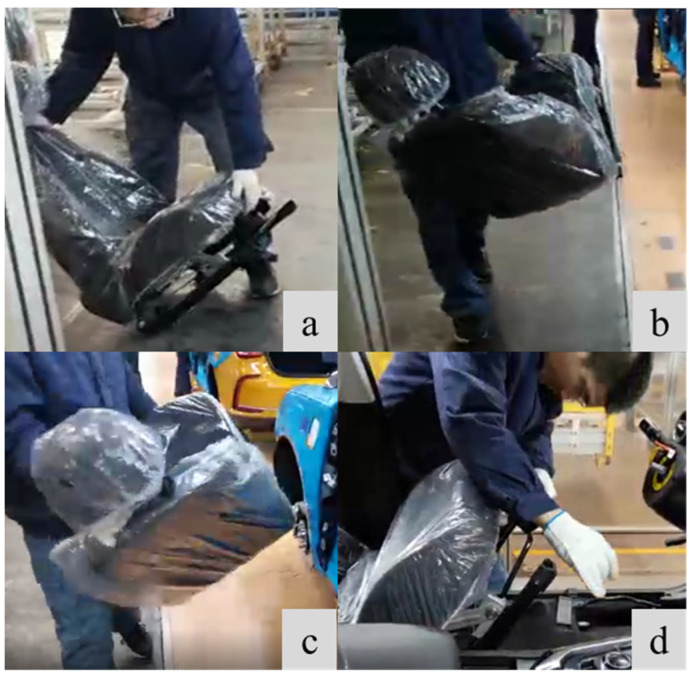
Car seat assembly process in a famous Chinese auto manufacturer: (**a**) Lifting start; (**b**) Lifting end; (**c**) Carrying stage; (**d**) Static stage.

This paper conducted a field investigation and in-depth research on a well-known Chinese auto manufacturer. In order to improve the accuracy of physical fatigue evaluation, considering the different action characteristics in the manual assembly process, the process in the automobile manual assembly line is divided into three stages: the lifting stage (Figure 1a,b), the carrying stage (Figure 1c), and the static stage (Figure 1d). The lifting stage mainly refers to the workers lifting the car doors, tires, seats, and other auto parts from the designated area; then workers carry these pre-assembled parts to the front of the auto body; finally, in this stage, workers need to bend over to operate, their torsos are static and only hands are performing fine operations, so this stage is called the static stage.

The main goal of this paper is to study a physical fatigue evaluation method for automobile manual assembly. This method combines the action characteristics of different stages in manual assembly, objective experimental data, and the characteristics of fatigue accumulation over time. It pays more attention to the dynamic behavior of workers and makes a more comprehensive and accurate physical fatigue evaluation of procedural behavior. To achieve this, we address the following issues:

- Can brain oxygen saturation represent the degree of fatigue in the human body?
- Do different carrying weights and attitude holding heights affect the degree of fatigue?
- Which of the fatigue evaluation methods proposed in this paper and energy expenditure in Tecnomatix Jack can better reflect fatigue?

## 2. Methods

### 2.1. Fatigue Evaluation Method

The process of automobile manual assembly is divided into three stages: the lifting stage, the carrying stage, and the static stage. Therefore, the appropriate ergonomic methods should be selected for these stages. The NIOSH 1991 (National Institute for Occupational Safety and Health) equation is selected for the lifting stage and the lifting index (LI) is used to represent the value of NIOSH 1991 [18,19]; Ovako Working Posture Analysis System (OWAS) is selected for the carrying stage, in order to reflect the effect of the quality of the object being handled on physical fatigue, a single digit was added to describe the weight to the three-digit version of OWAS proposed by Karhu et al. in 1977. The four digits are used for upper limb states, lower limb states, back states, and weight-bearing, respectively [20,21]; the Rapid Upper Limb Assessment (RULA) is selected for the static stage, which mainly involves the assembly details of the worker’s upper limbs [22,23]. These three methods are commonly used in the assessment of the risk of musculoskeletal injuries, whereas incorrect posture and repetitive high-load work resulting in energy depletion and rapid physical fatigue are the key factors leading to musculoskeletal injuries, and therefore these three methods can be used to assess the physical fatigue.

To establish a quantitative method of physical fatigue for automobile manual assembly, it is first necessary to determine the weights of NIOSH, OWAS, and RULA. The entropy weight method is used to calculate the weight of the above three methods. The entropy weight method calculates weights based on objective data changes [24], which can avoid the limitations and constraints of subjective conditions brought about by the weights given by methods, such as the analytic hierarchy process, based on personal experience and subjective judgment of evaluation experts [25,26]. For the index $x_{i}$, the greater the difference between the values $x_{ij}$ of the experimental data under the index, the greater the role of the index in the comprehensive evaluation [27]. Thus, the weights corresponding to NIOSH, OWAS, and RULA are, respectively w_1_, w_2_ and w_3_.

Then, the values of NIOSH, OWAS, and RULA are normalized and the changes of these values are fixed at [0, 1] without changing the original distribution of the data. The formula is as follows,

(1) $$r_{ij}=\frac{r_{ij}^{’}-Min(r_{ij}^{’})}{Max(r_{ij}^{’})-Min(r_{ij}^{’})},$$

Among them, $r_{ij}^{’}$ is the actual score during the experiment of NIOSH, OWAS, and RULA, and $r_{ij}$ is the normalized result of this value.

On this basis, because fatigue is a dynamic and cumulative process over time, the time-weighted method is used to weigh the actual duration of the lifting stage, the carrying stage, and the static stage, so that the final quantitative value of fatigue is

(2) $$e_{j}=\frac{\sum_{i=1}^{3}{r_{ij}w}_{i}t_{i}}{\sum_{i=1}^{3}t_{i}}$$

Among them, $e_{j}$ is the fatigue quantification value corresponding to group j experiments. w_i_ (*i* = 1, 2, 3) is the weight values of three indexes: NIOSH, OWAS, and RULA, respectively, and t_i_ (*i* = 1, 2, 3) is the actual duration of the three stages in each group of experiments: lifting, carrying, and static stage. Therefore, the construction of the physical fatigue evaluation method for automobile manual assembly is completed.

### 2.2. Experimental Design

#### 2.2.1. Experimental Hypothesis

The independent variables in this experiment are the seat weight and the height of the car body from the ground. The seat weights were, respectively 8 kg, 14 kg and 20 kg. The heights of the car bottom from the ground were selected as 15 cm and 45 cm. The dependent variable of the experiment was the level of physical fatigue of the manual assemblers. The level of physical fatigue is represented by three indicators, which are the difference of cerebral oxygen saturation, the value of physical fatigue evaluation method and metabolic energy expenditure. On this basis, the hypothesis of the experiment is that different seat weights and the height of the vehicle bottom from the ground have an effect on the level of physical fatigue.

#### 2.2.2. Experimental Manual Assembly Task

All experiments were carried out in the morning to ensure the consistency of the participants’ physical condition to a large extent, and each experiment was only performed once a day for each participant. The experimental site was in an ergonomic laboratory with good sound insulation to reduce the interference of external noise to participants. The laboratory was spacious and ventilated, with good lighting conditions and a suitable temperature to ensure the comfort and stability of the experimental environment. This experiment took the car seat assembly in the vehicle manual assembly as an example. The car seats were objects of equal quality, and the vehicle body was a virtual model displayed through the ARE platform. The distance between the seat and the vehicle body was kept consistent with the actual industrial site distance of 3 m. This experiment was an intra-group experiment, which was divided into 6 group experiments, with 3 participants in each group. First, participants needed to lift an object with the same weight as the seat at the starting point; then, the participants carried the object 3 m to the virtual vehicle body model; finally, they bent over to assemble the object into the correct position on the vehicle body, during which time their bodies remain static and only their hands can perform detailed operations for 10 s. The duration of each experiment was 30 min.

### 2.3. Apparatus and Platform

#### 2.3.1. Cerebral Oximeter

**Figure 2:**
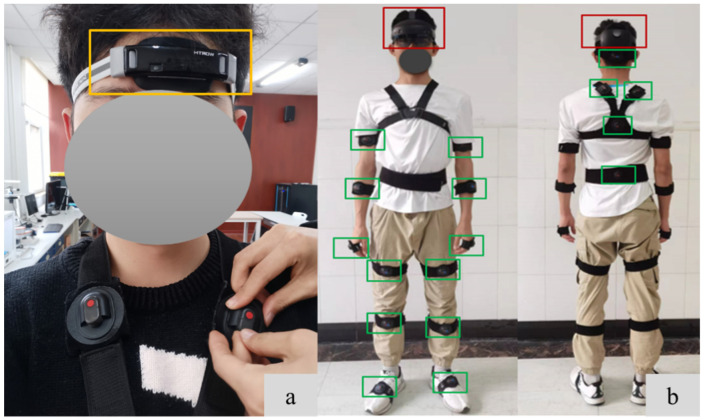
(**a**) Cerebral oximeter (yellow box); (**b**) Augmented reality devices (red box) and motion capture systems (green boxes).

The near-infrared tissue blood oxygen nondestructive monitor used in this experiment is wearable wireless oxygen saturation monitoring on the head (WORTH) (Figure 2a). The device is a wireless monitoring device independently developed by the Brain Network Group Research Center of the Institute of Automation, Chinese Academy of Sciences, which is founded in Beijing in 2017. A highly integrated central block is embedded in the device body, which includes an optical module, a microprocessor unit, a wireless communication module, and a power management module. Moreover, WORTH has the accuracy of recording cerebral oxygen saturation with an accuracy comparable to that of current clinically used cerebral oxygen monitors and has proved that it is effective during exercise tasks [28].

#### 2.3.2. Experiment Platform

The construction of the experimental environment and the calculation of ergonomic indexes (NIOSH, OWAS, RULA) were completed by the Augmented Reality (AR)-based Ergonomic Platform (ARE Platform) [29]. The ARE platform can put virtual manufacturing models in the physical environment based on the AR device, providing the participants with a semi-immersive working experience and retaining the constraints of the physical environment, thus completing the environment for fatigue experiments. Moreover, the ARE platform uses the motion capture system to collect a set of ergonomics indexes (NIOSH, OWAS, RULA), accessibility, and visibility verification data, which meets the real-time data required (NIOSH, OWAS, RULA) for the fatigue evaluation method. In addition, the accuracy of the ergonomics indexes provided by the ARE platform has been verified.

1. A commercial AR device Microsoft Hololens2 (Hololens2) (Figure 2b) is used for this platform. The total weight of Hololens2 is 566 g. The setup is equipped with four visible light cameras, two infrared cameras, a 1-MP time-of-flight depth sensor, and an inertial sensor. HoloLens2 can provide users with hand tracking, eye tracking, voice commands, spatial mapping, mixed reality capture, 6 degrees of freedom tracking, and other functions;
2. The motion capture system used in this platform is the Noitom Perception Neuron 3 (PN3) (Figure 2b), which can collect and process human posture data in real-time. PN3 includes 17 inertial sensors and the size of each inertial sensor is 27.9 × 16.2 × 11.6 mm, and the weight is 4.1 g, which makes users feel lighter and more flexible. It provides a data output frame rate of up to 60 Hz, and the static attitude accuracy is Roll/Pitch 1°, Yaw 2°. Moreover, PN3 connects the sensor data with computers through the Type-C interface, and the transmission delay is within 20 ms.

### 2.4. Participants

Three adult male participants were recruited to participate in this laboratory study (the seat assembly workers in the actual work site are men, so men were selected as subjects), and all participants were healthy without muscle strain. Participation was voluntary, with written consent, and anonymous. The ages of the participants ranged from 23 to 24 years (mean = 23.67 years), with average height and weight of 176.3 cm and 68.28 kg. Before the experiment, the participants were required to ensure adequate sleep, avoid any food and drugs that stimulate or inhibit the central nervous system, including coffee, alcohol, and tea, and avoid strenuous exercise. Before the start of the experiment, each participant was required to fully understand the entire experimental procedure, requirements, and testing methods, and to be proficient in the required work.

### 2.5. Outcome Measures

#### 2.5.1. Cerebral Oxygen Saturation

Cerebral oxygen saturation was chosen to represent fatigue in the experiment because changes in cerebral blood oxygen saturation are closely related to the cerebral circulation. Disease or physical activity can affect cerebral circulation, thereby altering cerebral blood oxygen saturation [30]. During intense exercise, hyperventilation reduces the carbon dioxide tension in the arteries and slows blood flow to the brain, which can lead to insufficient oxygen delivery to the brain, leading to the development of fatigue [31]. And the lack of oxygen in the brain causes a decrease in the cerebral oxygen saturation. This experiment uses ${rSO}_{2}$ to represent fatigue, which is the weighted average of cerebral arterial, capillary, and venous oxygen saturation [32,33]. In actual monitoring, ${rSO}_{2}$ is generally defined as the percentage of oxygen carried by hemoglobin at the target monitoring point, and its formula is

(3) $${rSO}_{2}=\frac{C_{{HbO}_{2}}}{C_{{HbO}_{2}}+C_{HbR}}\times 100\%$$

Among them, $C_{{HbO}_{2}}$ is the concentration of oxyhemoglobin; $C_{HbR}$ is the concentration of reduced hemoglobin, which refers to the content of the corresponding type of hemoglobin in the unit volume of blood.

The fluctuations of the cerebral oxygen saturation of the three participants in a calm state were detected many times, and the fluctuations of the cerebral oxygen saturation within 40 min were all between −0.5% and 0.5%. Therefore, it is assumed that the cerebral oxygen saturation did not change within 40 min of the three participants in a calm state.

#### 2.5.2. Physical Fatigue Evaluation Method

Using PN3, 17 inertial sensors were placed at specific joints of the body (Figure 2b). After the human body pose was calibrated, the kinematics data were collected and transmitted to the ARE platform for processing. After participants began lifting objects, the NIOSH score and lifting time were recorded; in the carrying stage, the OWAS score and carrying time were recorded; in the static stage, the RULA score was recorded and the default duration was 10 s. After the experiment, the NIOSH, OWAS, and RULA scores recorded were processed with the entropy weight method to obtain their respective weights. Next, the NIOSH, OWAS, and RULA scores were normalized according to Formula (1). Finally, according to the actual experimental duration of the three stages, Formula (2) was used to get the final quantitative value of the physical fatigue in each experiment.

#### 2.5.3. Jack MEE

The limitation of energy supply is the classic hypothesis of muscle fatigue, whereby energy deficit is an important factor in fatigue [34]. Tecnomatix Jack software, a module from the Siemens PLM digital factory portfolio, is a tool in the field of human factors reliability [35]. Tecnomatix Jack provides some advanced analysis tools for ergonomic analysis. Metabolic Energy Expenditure (MEE) is based on the defined human virtual model and divides typical tasks into 25 task behaviors such as lifting, carrying, sitting, standing, walking, and bending. On this basis, index parameters such as the working cycle time of the current task, specific working posture, and task load are set to predict the metabolic energy consumption of the current task.

Firstly, virtual human models were established in Tecnomatix Jack based on the height and weight of participants. On this basis, the proportion of each working posture was calculated according to the duration of different working postures in the experiment. The duration of each experiment was 30 min. According to the total number of times the participants performed under the conditions of different vehicle body heights and seat weights, the cycle time for completing a task can be calculated. According to the actual seat assembly workflow and the settings of this experiment, the lifting stage corresponds to the lifting task type in the MEE tool, the lifting stage corresponds to the handling task type in the MEE tool, and the static stage corresponds to the low position task type in the MEE tool. The energy consumption formulas of the working posture are as follows,

(4) $$E_{pos}=K_{pos}\times T_{pos}\times W$$

(5) $$E_{T}=\sum_{i=1,\ldots ,n}\;E_{i}+E_{\mathrm{standing}}+E_{\mathrm{sitting}}+E_{\mathrm{benting}}$$

Among them, $K_{pos}$ represents the energy consumption coefficient of different working postures, $T_{pos}$ represents the duration of the current posture, and W is the weight of the current operator. $E_{i}$ is the energy consumption of each action, where i represents the specific number of the action sequence, and $E_{T}$ represents the total energy consumption for completing the entire task.

### 2.6. Experimental Procedures

This experiment strictly followed the experimental ethics code, requiring participants to participate voluntarily after knowing the purpose of the experimental data. Firstly, participants practiced until they became familiar with the seat assembly process, with an average practice time of about 5 min. Then, they needed to wear the WORTH correctly for about 10 min and be in a calm state during this period, avoiding intense exercise or intense emotions to ensure the stability and accuracy of cerebral oxygen saturation data.

**Figure 3:**
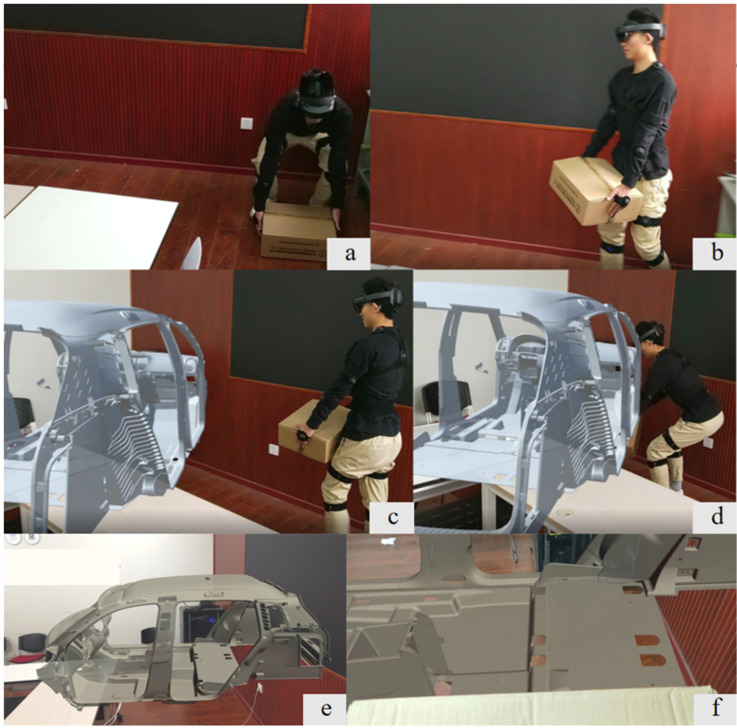
(**a**) Lifting start; (**b**) Lifting end; (**c**) Carrying stage; (**d**) Static stage; (**e**) First view of lifting end; (**f**) First view of static stage.

After taking off WORTH, participants needed to wear the PN3 motion capture system. They chose to use a strap of appropriate length to choose the position with the least amount of muscle to wear, and inserted PN3 sensors into the base of the strap to ensure that they would not fall off due to activities. Due to the variability of wearers and the inconsistency of each wearing part, it was necessary to calibrate participants’ posture data. Then, participants put the HoloLens2 on the head and adjusted the knob on the back of the device to adjust the tightness to ensure that it was comfortable to wear and did not shake with the head movement. After wearing HoloLens2 participants could see the virtual vehicle body model placed 3 m away. First, participants lifted the seat equivalent of a specific weight (8 kg, 14 kg, 20 kg); secondly, they carried the equivalent and walked 3 m to the virtual vehicle body model; finally, they placed the object on a specific vehicle body height (15 cm, 45 cm), and simulated 10 s of static assembly operation, and repeated the above operation until the 30 min mark (Figure 3). The MEE tool in Tecnomatix Jack performs simulations that do not involve detailed operations of the upper limbs, therefore boxes with the same quality as the three weights of seats were chosen for the experiment, thus comparing the method proposed in this paper with the results of MEE tool in Tecnomatix Jack performs simulations.

After the experiment was completed, participants took off the HoloLens2 and wore the WORTH again for about 10 min, and remained in a calm state within ten minutes, avoiding intense exercise or intense emotions. Then, they removed the WORTH and PN3 motion capture system.

### 2.7. Statistical Data Analysis

The dependent variable is the human physical fatigue level, which is represented by three indicators, which are the experimental results of the physical fatigue evaluation method and the difference of cerebral oxygen saturation. On this basis, the MEE simulation in Tecnomatix Jack was carried out for each group. Before the statistical analysis, the normality of the experimental results of physical fatigue evaluation method, the difference of cerebral oxygen saturation, and the simulation results of Jack MEE were tested by Shapiro–Wilk, and they all conformed to the normality. Then, the experimental results of the physical fatigue evaluation method, the difference of cerebral oxygen saturation, and the simulation results of Jack MEE were analyzed by two-way analysis of variance (ANOVA). The independent variables are (1) Seat weight: 8 kg, 14 kg, 20 kg, and (2) Vehicle body height: 15 cm, 45 cm. The statistical significance of ANOVA was *p* ≤ 0.05, and if statistical significance was found, a Pearson correlation analysis was performed to further clarify the differences between techniques.

## 3. Results

### 3.1. Cerebral Oxygen Saturation

The ${rSO}_{2}$ of each participant was measured for 10 min by the WORTH before and after the experiment, and selected the stabilized data from the monitored values as the baseline data for the subjects. According to the ${rSO}_{2}$ difference after and before the experiment in 18 groups, the results are shown in Table 1 below.

According to the experimental hypothesis, the hypothesis made on the height of the vehicle body from the ground and the seat weight are as follows,

$H_{0A}$ **:**
*Different vehicle body heights (15 cm, 45 cm) have no significant effect on the* ${rSO}_{2}$ *difference;*

$H_{1A}$ **:**
*Different vehicle body heights (15 cm, 45 cm) have a significant impact on the* ${rSO}_{2}$ *difference.*

$H_{0B}$ **:**
*Different seat weights (8 kg, 14 kg, 20 kg) have no significant effect on the* ${rSO}_{2}$ *difference;*

$H_{1B}$ **:**
*Different seat weights (8 kg, 14 kg, 20 kg) have a significant impact on the* ${rSO}_{2}$ *difference.*

Therefore, the two-way ANOVA was performed on the data in Table 1, and the following are the analysis results. A two-way ANOVA demonstrated that the effect of vehicle body height was significant for ${rSO}_{2}$ difference, F (1, 2) = 29.623, *p* < 0.001. And the effect of seat weight was significant for ${rSO}_{2}$ difference, F (2, 2) = 224.652, *p* < 0.001.

### 3.2. Physical Fatigue Evaluation Method

Firstly, the entropy weight method was used to process the 18 groups of NIOSH, OWAS, and RULA scores collected during the experiment (as Table 2), to obtain the respective weights, as shown in Table 3 below.

Then, the NIOSH, OWAS, and RULA values were normalized according to Formula (1). Finally, based on the specific duration data of the experiment and the Formula (2), the results obtained by the physical fatigue evaluation method are shown in Table 4. In order to ensure the accuracy of the subsequent comparison with the calculation results of the MEE tool, the calculation results of the physical fatigue evaluation method are not rounded.

According to the experimental hypothesis, the data in Table 3 were subjected to two-way ANOVA, and the results are as follows. A two-way ANOVA demonstrated that the effect of vehicle body height was significant for physical fatigue evaluation method results, F (1, 2) = 15.309, *p* = 0.002. And the effect of seat weight was significant for physical fatigue evaluation method results, F (2, 2) = 88.684, *p* < 0.001.

After that, a Pearson correlation analysis was used to analyze the correlation between the ${rSO}_{2}$ difference and the physical fatigue evaluation method results, and the correlation coefficient is obtained as shown in Table 5.

According to Table 4, we found a strong correlation between ${rSO}_{2}$ difference and physical fatigue evaluation method, r = −0.938, *p* < 0.001.

### 3.3. Jack MEE

After filling in the parameters such as task type and load in the MEE interface according to the experimental data, 18 sets of metabolic energy consumption (kcal) consumed by the experiment are shown in Table 6.

According to the experimental hypothesis, the data in Table 5 were subjected to two-way ANOVA, and the results are as follows. A two-way ANOVA demonstrated that the effect of vehicle body height was significant for metabolic energy expenditure, F (1, 2) = 6.525, *p* = 0.023 < 0.05. And the effect of seat weight was significant for metabolic energy expenditure, F (2, 2) = 125.845, *p* < 0.001.

After that, a Pearson correlation analysis was used to analyze the correlation between the ${rSO}_{2}$ difference and metabolic energy expenditure, and the correlation coefficient is shown in Table 7.

According to Table 7, we found a strong correlation between ${rSO}_{2}$ difference and metabolic energy expenditure, r = −0.924, *p* < 0.001.

## 4. Discussion

**Figure 4:**
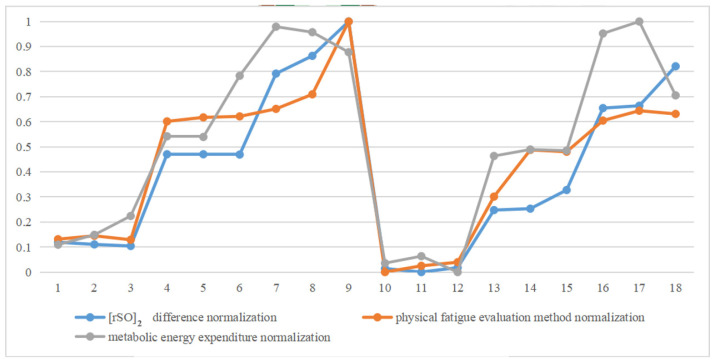
Normalization of ${rSO}_{2}$ difference, normalization of physical fatigue evaluation method, and that of metabolic energy expenditure in 18 groups of experimental trends comparison.

This paper systematically studied the effects of different seat weights and vehicle heights from the ground on physical fatigue during the manual seat assembly. The main research results showed that when the vehicle height was 15 cm and the seat weight was 20 kg, the physical fatigue level was the highest. On the contrary, when the vehicle height was 45 cm and the seat weight was 8 kg, the physical fatigue level was the lowest. The cerebral oxygen saturation was used as the objective physiological signal for fatigue detection in the experiment, and the correlation between the proposed physical fatigue evaluation method and the ${rSO}_{2}$ difference was slightly higher than that between the metabolic energy expenditure in Jack and the ${rSO}_{2}$ difference, which means the proposed physical fatigue evaluation method is more accurate than the evaluation of metabolic energy consumption in Tecnomatix Jack (Figure 4). The horizontal axis presents the numbers of the eighteen sets of experiments, and the vertical coordinates are the normalized values of the three fatigue evaluation methods.

### 4.1. Cerebral Oxygen Saturation

According to Table 1, when the seat weight was 8 kg and 14 kg, the impact of the vehicle height on the ${rSO}_{2}$ difference was more obvious than when the seat weight was 20 kg. When the vehicle height was constant, the influence of different seat weights on the ${rSO}_{2}$ difference was obvious. In Table 2, the results of ANOVA between vehicle height and seat weight on ${rSO}_{2}$ difference can show that these two independent variables both have a significant impact on ${rSO}_{2}$ difference.

### 4.2. Physical Fatigue Evaluation Method

According to the weights of NIOSH, OWAS, and RULA in Table 3, the degree of variation in the scores of NIOSH and OWAS among the experiments was greater than that of RULA. It showed that the influence of the vehicle height on the results of the physical fatigue evaluation method was less significant than that of the seat weight. In the two-way ANOVA results in Table 5, the vehicle body height and seat weight were significant to the results of the physical fatigue evaluation method. However, the significance of vehicle height to the results of physical fatigue evaluation method was weaker than its significance to ${rSO}_{2}$ difference. It showed that the physical fatigue evaluation method was slightly less sensitive to the vehicle body height. In Table 6, the correlation coefficient between ${rSO}_{2}$ difference and the results of the physical fatigue evaluation method was −0.938, and the *p* value was less than 0.05, indicating that there was a strong correlation between ${rSO}_{2}$ difference and the physical fatigue evaluation method. Because ${rSO}_{2}$ difference can represent the level of human physical fatigue, the physical fatigue evaluation method can reflect human physical fatigue level to a large extent.

### 4.3. Jack MEE

The *p* value of the vehicle body height to the metabolic energy expenditure from Jack MEE was 0.023. The *p* value was less than 0.05, the vehicle body height had a significant impact on the Jack MEE simulation results, but compared with the ${rSO}_{2}$ difference and physical fatigue evaluation method, the significant impact was smaller. Obviously, the sensitivity of the Jack MEE simulation to the vehicle body height was lower than that of the cerebral oxygen saturation signal and the physical fatigue evaluation method. According to the Pearson correlation analysis results, it can be concluded that there was a strong correlation between the Jack MEE simulation results and the ${rSO}_{2}$ difference, while the correlation coefficient was −0.924. However, the correlation coefficient between the results of the physical fatigue evaluation method and the ${rSO}_{2}$ difference was −0.938, which was closer to −1 than the difference between metabolic energy expenditure and ${rSO}_{2}$. Therefore, the physical fatigue evaluation method, to a certain extent, can more accurately reflect the level of human physical fatigue than the metabolic energy expenditure prediction in Tecnomatix Jack, and was more sensitive in maintaining the height of posture in the static stage.

## 5. Conclusions

This paper conducts an in-depth study on the evaluation method of human physical fatigue, oriented by the practical problems in the manual assembly of automobiles. This paper divides the automobile manual assembly process into three stages: the lifting stage, the carrying stage, and the static stage, and uses NIOSH, OWAS, and RULA to analyze these stages, respectively. Moreover, the ARE platform provides an AR environment and the calculation of ergonomic indexes (NIOSH, OWAS, and RULA) for the experiment. On this basis, the entropy weight method and time weighting are used to process the experimental data to complete the physical fatigue evaluation method. And using the MEE tool in Tecnomatix Jack to simulate and predict the metabolic energy expenditure in the experiment. In this paper, the vehicle seat assembly in the auto manual assembly is taken as an example to carry out experiments. The independent variables are seat weight and vehicle body height from the ground, and the dependent variable is the human physical fatigue level. Eighteen groups of effective experiments have been completed. Finally, this paper conducts a Pearson correlation analysis on the physical fatigue evaluation method and the difference between cerebral oxygen saturation and metabolic energy expenditure in Tecnomatix Jack. The results prove that compared to the metabolic energy expenditure tool in Tecnomatix Jack, the physical fatigue evaluation method refines and summarizes the entire dynamic manual assembly process of automobiles to obtain a more accurate physical fatigue evaluation level, which is exactly the innovation of this article. This provides a new ergonomic solution for intelligent manufacturing and provides a reference and support for the future development direction of human fatigue evaluation. In recent years, more and more researchers have made new explorations in the direction of physical fatigue evaluation. Just as the method used in this paper, there are many cases of using wearable devices for fatigue evaluation, and most of them have also achieved good results [36,37,38]. The use of wearable devices has the advantages of high precision, high flexibility and high adaptability. In the future it may become the primary means of accurately assessing physical fatigue.

However, some aspects of the current research content of this paper still need to be further studied. In the current physical fatigue evaluation method, the worker’s posture is approximated as a static action during the last stage of assembly. But in fact, the workers’ fingers still perform assembly movements. Therefore, in future research, the upper limbs should be decomposed and analyzed in more detail to improve the accuracy of the fatigue evaluation method. Because the number of experiments in this study is limited, it is necessary to increase the number of experiments, further explore the characteristics of experimental data, and improve the accuracy of the fatigue evaluation method in future research, while enabling the Physical Fatigue Evaluation Method to give a specific fatigue level. Moreover, this paper does not discuss the impact of the load brought by AR to the experiment in the physical fatigue evaluation, so it can be studied in future research on fatigue evaluation in the AR environment.

## Figures and Tables

**Table 1:** The change value of ${rSO}_{2}$ before and after the experiment.

	Seat Weight (kg)	8	14	20
Vehicle Body Height (cm)				
15		−4.26% ± 0.5%	−9.92% ± 0.5%	−15.11% ± 0.5%
		−4.12% ± 0.5%	−9.92% ± 0.5%	−16.25% ± 0.5%
		−4.02% ± 0.5%	−9.9% ± 0.5%	−18.46% ± 0.5%
45		−2.57% ± 0.5%	−6.33% ± 0.5%	−12.89% ± 0.5%
		−2.34% ± 0.5%	−6.42% ± 0.5%	−13.04% ± 0.5%
		−2.63% ± 0.5%	−7.62% ± 0.5%	−15.58% ± 0.5%

**Table 2:** The value of NIOSH, OWAS, and RULA.

Experiment No.	Value of NIOSH	Value of OWAS	Value of RULA
1	0.46	1	6
2	0.59	1	6
3	0.43	1	6
4	1.1	3	6
5	1.28	3	6
6	0.93	3	7
7	1.88	3	5
8	2.01	3	6
9	2.51	4	7
10	0.59	1	3
11	0.42	1	4
12	0.51	1	4
13	0.9	2	4
14	0.95	3	4
15	0.87	3	4
16	1.83	3	4
17	1.94	3	4
18	2.18	3	5

**Table 3:** Weights of NIOSH, OWAS, and RULA.

Evaluation Method	Information Entropy Value *e*	Information Utility Value *d*	Weights (%)
NIOSH	0.854	0.146	40.616
OWAS	0.852	0.148	41.194
RULA	0.935	0.065	18.19

**Table 4:** The results obtained by the physical fatigue evaluation method.

	Seat Weight (kg)	8	14	20
Vehicle Body Height (cm)				
15		0.010766997	0.019983377	0.020962028
		0.011034003	0.020297028	0.022100687
		0.01072171	0.020373405	0.027797152
45		0.008193415	0.014098114	0.020047302
		0.008682337	0.01775242	0.020818436
		0.008965525	0.017608327	0.020569058

**Table 5:** Correlation analysis results of ${rSO}_{2}$ difference and physical fatigue evaluation method.

Variable	${rSO}_{2}$ Difference	Physical Fatigue Evaluation Method Results
${rSO}_{2}$ Difference	1 (<0.001)	−0.938 (<0.001)
Physical Fatigue Evaluation Method Results	−0.938 (<0.001)	1 (<0.001)

**Table 6:** The metabolic energy expenditure (kcal) in Tecnomatix Jack.

	Seat Weight (kg)	8	14	20
Vehicle Body Height (cm)				
15		1267.80	1575.37	1886.35
		1295.80	1573.48	1870.81
		1349.33	1747.11	1814.05
45		1215.47	1519.52	1867.15
		1235.22	1537.95	1901.10
		1190.07	1535.00	1691.50

**Table 7:** Correlation analysis results of ${rSO}_{2}$ difference and metabolic energy expenditure.

Variable	${rSO}_{2}$ Difference	Jack MEE Results
${rSO}_{2}$ Difference	1 (<0.001)	−0.924 (<0.001)
Jack MEE results	−0.924 (<0.001)	1 (<0.001)

## Data Availability

In the informed consent form filled out by the subjects before the start of the experiment, it was written “Only the research team will have access to the data”. In order to protect the privacy of the subjects, we decided not to disclose the experimental data.
